# Persistent Mullerian Duct Syndrome in an Adult Infertile Male: A Case Report

**DOI:** 10.31729/jnma.8749

**Published:** 2024-09-30

**Authors:** Nesuma Sedhain, Shree Prasad Adhikari, Hema Kumari Pradhan, Rakshya Parajuli

**Affiliations:** 1 Department of Obstetrics and Gynecology, Paropakar Maternity and Womens' Hospital, Thapathali, Kathmandu, Nepal; 2 Department of Obstetrics and Gynecology, Kathmandu Model Hospital, Pradarsani Marg, Kathmandu, Nepal

**Keywords:** *anti-mullerian hormone*, *cryptorchidism*, *persistent mullerian duct syndrome*, *male infertility*, *orchidopexy*

## Abstract

Persistent Müllerian duct syndrome(PMDS) is a rare autosomal recessive disorder of sex development characterized by the presence of Müllerian duct derived structures in a normally virilized, genotypical (46, XY) and phenotypical male. Here we describe a case of a couple who presented as primary subfertility with azoospermia and diagnosed as PMDS (Bilateral testis in right inguinal canal with associated ring inguinal hernia and persistent of mullerian duct derivatives in the form of rudimentary uterus and fallopian tubes) and planned for Intrauterine insemination with donor semen. PMDS is rare disease and in developing countries like Nepal, because of unaware of the disease condition and lack of proper health care facilities, such cases are diagnosed later in adult males. Therefore, this case highlights the importance of awareness and knowledge for early detection and treatment of such conditions to conserve fertility and prevent malignancy of testis and other remnant mullerian structures.

## INTRODUCTION

Persistent Mullerian duct syndrome (PMDS) is a rare disorder occurring in men with a normal phenotype and genotype (46,XY) and is characterized by the presence of Mullerian duct structures like uterus, cervix, fallopian tubes and upper two thirds of vagina.^1^ Sex determination in male is regulated by testosterone and anti-mullerian hormone. PMDS arise due to failure of mullerian regression, cause of which may be Idiopathic or mutation in activating anti-mullerian Hormone (AMH) or its receptor AMHRII. Thus mullerian duct derivatives remains along with testis and male excretory ducts and the external virilization is complete.^2,3^ Here we present a case of PMDS who presented with infertility.

## CASE REPORT

A 31 years female with her male partner 39 years, presented in our OPD with primary subfertility even after 14 years of consummative marriage. The couple had two failed cycles of Intrauterine insemination (IUI) with donor semen in view of azoospermia at other center. However, evaluation for the cause of azoospermia was not done. Both the partners were reevaluated, semen analysis showed azoospermia however investigations of female partner were normal.

All his three siblings were healthy and had children. On further examination, BMI: 28 kg/m^2^, blood pressure: 110/70 mm/Hg, normal male pattern pubic and axillary hair distribution, no gynecomastia. On genital examination, penis and urethra were normal. Left testes was not palpable in scrotum. A firm, nontender swelling around 5x3 cm was palpable in right inguinoscortal region.

**Figure 1:**
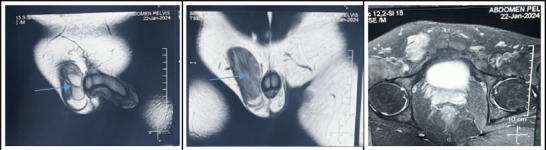
MRI image, arrow shows both testis in right inguino scrotal sac (A); Right inguinal defect through which omentum is herniating (B); rudimentary uterus and fallopian tubes lying behind the bladder (C).

On ultrasonography of abdomen, pelvis and scortum; bilateral scortum was empty with both testes located in right inguinal region and bilateral hydrocele. MRI abdomen and pelvis report suggested features probable of persistent mullerian duct syndrome as Bilateral testis in right inguinal canal with associated ring inguinal hernia. Transverse testicular ectopia showed by persistent mullerian duct derivatives in the form of rudimentary uterus and fallopian tubes and horse shoe shaped kidney (Figure 1A, 1B, and 1C).

Blood hormone assay showed testesterone: 2.27 ng/ml (1.75-7.81), Follicular stimulating hormone (FSH) : 18.7 μIU/ml (1.55-9.74), Leutenizing hormone (LH) : 6.56 mlU/ml, Inhibin B : 22 pg/ml (134.7-150.5 pg/ml), AMH level was low, i.e. 0.08 (0.7-16ng/ml), Alpha fetoprotein (AFP) : 4.08 ng/ml (<7.51), PHCG: <2.39. Karyotyping showed male genotype (46,XY).

He was referred to urologist where diagnostic laparoscopy was planned but patient refused. Couple was also given option of testicular biopsy and spermatozoa retrieval followed by in vitro fertilization and intracytoplasmic sperm injection (IVF-ICSI). However they refused and opted for donor Intrauterine Insemination (IUI).

## DISCUSSION

PMDS is a rare variant of male pseudohermaphroditism, where genotypically male individual has testis and the structures derived from both Mullerian and Wolffian ducts. Around 300 cases of PMDS have been described.^4^ They are commonly inherited through autosomal recessive pattern, however some have autosomal dominant and X-linked pattern. About 84% PMDS cases are caused by mutation in AMH gene (on the short arm of chromosome 19) and AMHR^2^ gene (on the long arm of chromosome 12). However, mutation are not detected in about 16% of cases.^3^ In PMDS there is either failure of synthesis or release of MIF or defect in end organs or error in the timing of release of MIF before the 8th gestational weeks results in persistence of mullerian structures. Since testosterone levels are normal, the Wolffian duct development takes a normal path and external genitalia are normal.^5^ However, the vas may be abnormal, narrow, blind, or even absent. Epididymal dissociation from the testis is common.^2^

**Three main clinical presentations are:**

1. Bilateral cryptorchidism: female variant (60-70%) where the testes are located in the pelvis, in the position of normal sized ovaries.
2. Unilateral cryptorchidism: male variant (20-30%) where one testis is in an inguinal hernia along with its attached tube and uterus, also known as hernia uteri inguinalis.
3. Transverse testicular ectopia: male variant (10%) where both testes and part of the mullerian organs have herniated into a single processus vaginalis).^2^

This case presented is of transverse testicular ectopia. Males with PMDS usually presents incidentally with bilateral undescended testes or inguinal hernia in childhood or sometimes in adult. They may even present with abdominal mass or testicular torsion, tumor or malignancy.^1^ Less frequent, but cases of malignancy of mullerian duct derivatives has also been reported.^6^ Some are even diagnosed while the couple undergo infertility assessment. Causes of infertility in PMDS are testicular hypoplasia, absent or abnormal vas and ejaculatory duct obstruction due to compression by Mullerian Duct structures. Fertility is rare but possible if at least one testis is scrotal and its excretory ducts are intact.^2,7^

Diagnose of PMDS is difficult, patients of any age group, who present with bilateral or unilateral undescended testis with one-sided inguinal hernia with palpable mass above the normally descended testis should ideally be investigated.^5^ Investigations includes ultrasonography, CT scan or MRI to assess the presence of uterus and fallopian tubes with testes.^8^ Karyotyping adds to confirm the diagnosis. AMH helps to identify the etiology. Cases with primary AMH receptor mutations have normal AMH level, while those with mutation of AMH gene as in this case it is very low or even undetectable.^9^

In PMDS orchidopexy with preservation of vas to maintain fertility is the main treatment of choice. However, in presence of streak gonads, or immobile testes, or any suspicion of malignancy then orchidectomy is done. Surgeries to remove the Mullerian duct structures are also performed, as they may later hypertrophy and congest causing pain and discomfort and rarely malignant transformation may occur. Sometimes they may compress the prostatic utricle causing recurrent urinary tract infection, stones and voiding difficulties.^8^ Complete excision of mullerian duct remnant can result in damage to the vascularity of the vas deferens as it is usually found adhered to lateral walls of the uterus, fallopian tubes, and vagina. Therefore, if the testes are normal, bilateral proximal salpingectomy, leaving fimbriae with epididymis along with subtotal hysterectomy and bilateral orchidopexy can be done. This helps in fertility preservation.^10^

Thus diagnosis and treatment of PMDS at early age can reduce the risk of malignancy and infertility. The main purpose of this study was to increase awareness among the health care providers and the parents about this rare disease.

## References

[1] Chua I, Samnakay N (2022). Persistent Müllerian Duct Syndrome: Understanding the Challenges.. Case Rep Urol.

[2] Picard JY, Cate RL, Racine C, Josso N (2017). The Persistent Müllerian Duct Syndrome: An Update Based Upon a Personal Experience of 157 Cases.. Sex Dev..

[3] Ahadi M, Soleimantabar H, Javanmard B, Zahedifard S (2020). A Rare Case of Persistent Mullerian Duct Syndrome and Review of Literature.. Int J Cancer Manag..

[4] Da Aw L, Zain MM, Esteves SC, Humaidan P (2016). Persistent Mullerian Duct Syndrome: a rare entity with a rare presentation in need of multidisciplinary management.. Int Braz J Urol..

[5] Agrawal AS, Kataria R (2015). Persistent Müllerian Duct Syndrome (PMDS): a Rare Anomaly the General Surgeon Must Know About.. Indian J Surg..

[6] Manjunath BG, Shenoy VG, Raj P (2010). Persistent müllerian duct syndrome: How to deal with the müllerian duct remnants - a review.. Indian J Surg..

[7] Mansour M, Fattal A, Ouerdane Y, Alsuliman T, Kanjawi O (2021). A 35-year-old father with persistent Mullerian duct syndrome and seminoma of the right undescended testis: a rare case report.. Surg Case Rep..

[8] Jeyakumar A, Ramachandran R, Rangasami R, Jeyakumar L, Gadupudi V (2022). Reviewing recherche presentations of persistent Mullerian duct syndrome: case reports.. Egypt J Radiol Nucl Med..

[9] Jandou I, Mhanna T, Chennoufi M, Aynaou M, El Houmaidi A, Barki A (2020). Hypofertility in a persistence of mullerian duct syndrome: Case report.. International Journal of Surgery Case Reports..

[10] Sankapal P, Gite VA, Agrawal M, Sane M, Singal A (2021). Persistent Mullerian Duct Syndrome: A Rare Case of an Adult Infertile Male with Bilateral Cryptorchidism.. J Reprod Infertil..

